# Crowdlending: mapping the core literature and research frontiers

**DOI:** 10.1007/s11846-021-00491-8

**Published:** 2021-11-10

**Authors:** Samuel Ribeiro-Navarrete, Juan Piñeiro-Chousa, M. Ángeles López-Cabarcos, Daniel Palacios-Marqués

**Affiliations:** 1 Universitat Politècnica de València, Valencia, Spain; 2 Santiago de Compostela University, Santiago de Compostela, Spain

**Keywords:** Crowdlending, P2P lending, Bibliometrics, G00, L26, M13

## Abstract

Peer-to-peer (P2P) lending uses two-sided platforms to link borrowers with a crowd of lenders. Despite considerable diversity in crowdlending research, studies in this area typically focus on several common research topics, including information asymmetries, social capital, communication channels, and rating-based models. This young research field is still expanding. However, its importance has increased considerably since 2018. This rise in importance suggests that P2P lending may offer a promising new scientific research field. This paper presents a bibliometric study based on keyword co-occurrence, author and reference co-citations, and bibliographic coupling. The paper thus maps the key features of P2P lending research. Although many of the most cited papers are purely financial, some focus on behavioral finance. The trend in this field is toward innovative finance based on new technologies. The conclusions of this study provide valuable insight for researchers, managers, and policymakers to understand the current and future status of this field. The variables that affect new financial contexts and the strategies that promote technology-based financial environments must be investigated in the future.

## Introduction

Crowdfunding is an innovative form of finance, and analysis of the factors that influence the performance of crowdfunding projects is necessary (Chen et al. 2020). The success of crowdfunding is built on the digitalization of society and the increasing presence of the Internet (Bouncken et al. 2015). This scenario requires knowledge and collaboration at all levels (Bouncken et al. 2021). Crowdfunding has gained in popularity since the 2008 financial crisis and is of particular relevance for small and medium-sized enterprises (SMEs), especially startups. Using platform mediation, crowdfunding gathers together small contributions from a large number of investors to finance projects with varying objectives (Cholakova and Clarysse 2015). Crowdfunding covers a wide range of approaches, including reward-based crowdfunding, equity crowdfunding, donation crowdfunding, and crowdlending. Crowdlending has received considerable attention from researchers in recent years (Bruton et al. 2015). This interest is expected to continue to intensify, hence the motivation for this paper.

Crowdlending transactions mainly take place in online environments, where lenders and borrowers engage in convenient, trustworthy exchanges. Peer-to-peer (P2P) lending involves platforms that join lenders and borrowers, who seek funding to carry out their projects. Accordingly, P2P lending platforms act as intermediaries between lenders and borrowers. They facilitate exchanges through credit screening services, posted interest rates (Wei and Lin 2017; Franks et al. 2021), default prediction (Franks et al. 2016), and formal and informal lending procedures (Allen et al. 2019).

One of the main concerns about P2P lending platforms is the need for all parties to have robust, accurate, timely information. To meet this need, borrowers and lenders use signals to transmit their intentions and positions in the lending market. Specifically, the better informed party uses signals to send information to the other party and thus aid the exchange between the two to make up for missing information. These signals overcome problems of information asymmetry that can arise with P2P transactions. To avoid information imbalances, these signals, in addition to attracting funding, must provide consistent and accurate information (Connelly et al. 2011), communicate abilities (Connelly et al. 2011), help build reputation (Walsh et al. 2015), generate credibility (Leischnig and Enke 2011), highlight the quality of offerings (Helm and Özergin 2015), and develop co-creative offerings (Patterson 2016). P2P lending transactions still face information asymmetry problems (Dorfleitner et al. 2016). Thus, lenders often have to make decisions based only on information published by borrowers without being sure about its authenticity (Klafft 2008a, b). The communication process, choice of communication channels, and organizational social capital can significantly influence the effect of signals in reducing or eliminating information asymmetries.

A growing stream of research has examined the role of social capital in facilitating (or hindering) economic exchanges (Granovetter 2005; Guiso et al. 2004). Social capital refers to features of social organizations such as networks, rules, and trust. These features develop from informal interactions or norms of universal reciprocity. They are then preserved by mutual commitment and cooperation (Putnam 1995; Adler and Kwon 2002). According to Granovetter (2005), social capital works best when created by the actions, patterns, or processes of those outside the economic setting in question. Thus, social capital can facilitate transactions with third parties outside the dyad that actually creates the social capital. Social connections can be beneficial because, in addition to conveying information flows between parties, the ties they generate offer a valuable cue for outsiders to infer the quality of the agents involved. In sum, by providing information and mutual benefits, social capital can mitigate potential inefficiencies caused by information imbalances, thus enhancing market efficiency (Durlauf and Fafchamps 2005). The negative implications of information asymmetries in the P2P lending market have led to the use of rating-based models to help evaluate and rank loans according to their risk or likelihood of default (Bastani et al. 2019). Together with risk variables, the evaluation process also considers loan returns. Ultimately, lenders face a multi-objective problem corresponding to traditional portfolio optimization (Deb et al. 2011). What is not so traditional is the context in which these financial transactions occur. Technology is revolutionizing P2P lending for two reasons. First, technology is increasingly present in the models (e.g., machine learning and neural networks) used to evaluate loans and thus mitigate information asymmetries in lending exchanges. Second, technology is also present in the process of P2P lending, namely in the way that P2P platforms operate (e.g., through blockchain or artificial intelligence).

This paper analyzes the P2P lending literature. Specifically, it examines why and how the concept of P2P lending has evolved from crowdsourcing and crowdfunding and explores which concepts or research lines are evolving and what direction the research will take in the future. To answer these questions, a bibliometric study was conducted. Bibliometric studies use statistical analyses of scientific publications (Pritchard 1969) to provide objective, impartial information on a specific field of research (Zupic and Čater 2015). To date, no bibliometric study has been conducted on this topic. Therefore, it is important to analyze the degree of research progress of P2P lending studies. This analysis can reveal the most important authors, references, journals, and keywords, together with the most relevant connections between them. As in recent previous research (Dana et al. 2021; Mas-Tur et al. 2020), bibliographic coupling, word co-occurrence, and co-citation analysis were used. Bibliometric methods were used to analyze the latest research on P2P lending and understand how this research topic has evolved and how it will continue to evolve in the future. Specifically, the study aims to describe the current research on P2P lending, identify the most relevant authors, publications, and journals, detect the most recurrent keywords from 2003 to February 2021, map the relationships between the key elements of this research field, and identify the fundamental topics at the P2P lending research frontier. In-depth analysis of previous findings in a particular field is necessary for that field to advance (Zupic and Čater 2015). Therefore, this bibliometric study contributes to building a complete picture of P2P lending research by considering all publications on the topic since its inception. The conclusions of the study can provide valuable insight for researchers to understand the current status and future of the field, for managers to search for new developments to improve performance and compete, and for policymakers to design strategies to promote growth and development.

The rest of the paper is organized as follows. Section 2 develops the conceptual framework. Section 3 describes the method. Section 4 presents and discusses the results. Section 5 indicates the limitations and practical implications. And finally, Sect. 6 shows the main conclusions as well as the future lines of research.

## Theoretical framework

### Crowdfunding

The phenomenon of the crowd (Franzoni and Sauermann 2014) has given rise to advances such as citizen science. This area considers the active participation of different individuals in scientific projects (Cappa et al. 2016, 2018). There is thus a need to assess the openness–performance relationship (Moretti and Biancardi 2020). The intersection between citizen science and the phenomenon of the crowd forms the basis of crowdfunding, which has become a prominent research topic. Crowdfunding is a type of outsourcing where funds are raised from a crowd of individuals. More specifically, crowdfunding refers to the use of a large number of individuals or groups to finance projects through small contributions pledged online without the need for standard financial intermediaries (Mollick 2014). Crowdsourcing allows organizations to externalize problem-solving tasks to obtain solutions from the crowd (Garcia Martinez 2015), whereas crowdfunding allows the crowd to play a complementary role, not only in the solution of tasks but also in the mobilization of capital (Ordanini et al. 2011). Pre-COVID-19, these transactions were already becoming increasingly important. However, the pandemic has forced organizations that used the Internet as a secondary business channel to prioritize innovative solutions using online platforms (Al-Omoush et al. 2020).

Crowdfunding has driven the democratization of the financial sector. It has helped promote entrepreneurial finance (Assenova et al. 2016; Block et al. 2018) and has connected lenders and entrepreneurs. The strength of crowdfunding lies in the widespread adoption and social acceptance of the Internet. This adoption and acceptance has created the framework to attract a multitude of online supporters and investors (Agrawal et al. 2015; Short et al. 2017). Using platform-mediated approaches, a large number of small investors can finance different types of projects—from nonprofit to innovative new ventures—that would otherwise not have had access to traditional financing. To initiate the process, entrepreneurs provide information to potential funders about the project through crowdfunding platforms. These platforms offer a marketplace where fund seekers can interact with crowds (Bruton et al. 2015). This information, which must capture funders’ attention, ranges from income figures or business plans to the aspirations and promises of entrepreneurs.

Crowdfunding projects differ depending on the potential reward or the motivations of entrepreneurs and funders. There are two main types of crowdfunding: profit-based crowdfunding and donation- or reward-based crowdfunding. In donation-based crowdfunding, contributors do not receive rewards, or if they do, these rewards are merely symbolic. The motivation of funders in this case is intrinsic (Gerber and Hui 2013). In reward-based crowdfunding, the motivation of funders may be both extrinsic, when they receive some reward, and intrinsic, when they receive nonpecuniary tangible (prototypes) or intangible (experiences) rewards in exchange for their support (Cholakova and Clarysse 2015). Within reward-based crowdfunding, Coakley and Lazos (2020) differentiated between equity crowdfunding and debt crowdfunding. In equity crowdfunding, funders receive shares or enter a revenue-sharing scheme in return for their contribution. The motivation is primarily extrinsic (Cholakova and Clarysse 2015; Colombo et al. 2015; Vismara 2018). In debt crowdfunding, also known as peer-to-peer (P2P) or marketplace lending, supporters receive interest as compensation for the risk and duration of their lending (Allison et al. 2013; Bruton et al. 2015). The motivation ranges from intrinsic to extrinsic depending on the financial returns (Ordanini et al. 2011). The complexity of the process and the need for investors to become involved are also criteria for classifying crowdfunding. Equity crowdfunding, lending crowdfunding, and reward-based crowdfunding are, in that order, the most complex forms of crowdfunding. As such, they require greater involvement. Conversely, donation crowdfunding involves less complexity and supporter involvement (Hornuf and Schwienbacher 2018).

Scholars have studied crowdfunding from different points of view, including crowdfunder motivation (Ordanini et al. 2011), crowdfunder types and definitions (Mollick 2014; Schwienbacher and Larralde 2012), signaling (Burtch et al. 2013), success factors and dynamic aspects (Kuppuswamy and Bayus 2017), the geographic distribution of investments (Agrawal et al. 2011), social capital (Lin et al. 2013; Mollick, 2014), local altruism and social capital (Giudici et al. 2018), communication (Courtney et al. 2017), narratives (Parhankangas and Renko 2017), fund seekers’ education, gender, and professional background (Barbi and Mattioli 2019), fund seekers’ social ties (Simon et al. 2019), professional funding (Roma et al. 2017), consumer perceptions (Wehnert et al. 2019), and science and technology (Colombo et al. 2015; Sauermann et al. 2019).

### P2P lending

The concept of P2P lending, similar to that of matchmaking (Evans and Schmalansee 2016), refers to operations through multi-sided platforms and virtual marketplaces to facilitate transactions between agents. The main objective of P2P markets is to engage buyers and sellers in convenient, trustworthy transactions. Although P2P lending is linked to finance, its increasing importance is due to the rise of matchmakers such as Amazon and eBay and the appearance of fintech startups such as Monzo and Funding Circle. This novel form of financing fits with the concept of ‘ingenious’ or creative solutions’ that combine novelty and value (Lampel et al. 2014). P2P lending involves a two-sided platform that brings together a crowd of individuals and borrowers (predominantly SMEs in need of credit). In exchange for a commission for hosting the lending campaign, crowdlending platforms offer SMEs the chance to obtain financing from investors who pay no commission and expect some kind of financial rewad. The reputation of P2P platforms in relation to credit risk assessment, together with the informal network of relationships among companies, can determine the ability of these companies to obtain P2P loans from a wide range of investors. Thus, active P2P platforms in the SME credit market act as intermediaries between investors (small investors and financial institutions) and firms, providing a wide variety of services such as credit screening (Wei and Lin 2017; Franks et al. 2021). There are indirect network externalities between small investors and financial institutions. Investments by institutions, which perform their own monitoring, provide a guarantee for the crowd. This guarantee helps reduce information asymmetries (Cumming and Hornuf 2020). Some authors claim that small investors tend to herd after institutional investors in equity-based crowdfunding. Similar strategies occur in P2P business lending (Asterbo et al. 2018). The crowd’s endorsement for campaigns, the so-called “wisdom of the crowd” (Mollick and Nanda 2016), can likewise act as an indirect network externality for institutions.

P2P marketplaces provide different sources of external debt finance. These sources range from P2P business loans to invoice finance. This form of financing habitually involves three agents: the funding platform, the borrowing firm (usually SMEs), and the crowd of investors. Although P2P lending is considered similar to bank lending, there are major differences regarding regulatory arbitrage and disintermediation (Coakley and Huang 2020). For instance, P2P loans are not subject to Basel III capital requirements. Therefore, P2P lending platforms have a relative advantage in terms of their lending rate when compared with commercial banks. In addition, for tax purposes, investors can offset bad loan losses with other crowdfunding income. Although some authors argue that P2P loans complement rather than compete with conventional banking (Milne and Parboteeah 2016), others argue that P2P lending has major advantages by combining the information advantages of informal lending with the pooling and risk-sharing benefits of financial intermediation (Allen et al. 2019). The main disadvantage of P2P loans versus bank deposits lies in their lack of protection.

### Information asymmetry

According to Spence (1973), signaling theory explains how signals help fill gaps in information between different parties by sending signals to make up for that missing information. The most informed party sends signals (observed variables) to the less informed party, disclosing the necessary information to make the exchange possible. Initially, this theory was applied to situations where there was little information on the credibility or quality of a product, service, or supplier (Kirmani and Rao 2000). It can now be applied to any situation where there is an information imbalance or information asymmetry between parties. Information asymmetry can lead parties to suffer adverse selection (Akerlof 1970). This adverse selection occurs when decision makers cannot observe or judge a situation based on the information provided (Pouryousefi and Frooman 2019). Ultimately, it can lead to financial losses (Petersen and Rajan 2002). Signals provide consistent information to all parties. These parties use this shared information (Connelly et al. 2011) to make transactions easier. Signals are useful for a wide variety of activities, most notably attracting financing (Ribeiro-Soriano et al. 2020).

Recently, the analysis of signaling and information asymmetries with respect to crowdfunding has captured the attention of researchers (Burtch et al. 2013; Courtney et al. 2017). This topic is highly relevant to crowdfunding, especially crowdlending (Ahlers et al. 2015; Courtney et al. 2017). P2P lending has a crucial difference with respect to the traditional financial credit market in that it involves no financial intermediaries (Lee and Lee 2012). These intermediaries are seen as repositories of soft information about credit quality (Petersen and Rajan 2002). For this reason, in P2P lending transactions, information is more difficult to verify, lenders are less sophisticated, the institutional framework is less developed, and information asymmetries frequently arise. In this context, signals are crucial for investors to reduce investment risk (Ahlers et al. 2015; Courtney et al. 2017). Borrowers signal and transmit information about themselves and the characteristics of the investment project, while lenders search for credit information and screen loan applicants (Yan et al. 2015).

In addition to signaling, there is still no clear evidence as to what other variables can improve overall trust in P2P lending projects. Trust management, which can significantly promote fundraising performance (Zheng et al. 2016), is crucial in financial contexts where the risk and complexity derived from economic transactions are important variables (McKnight et al. 1998). P2P lending involves financial transactions that are extremely trust intensive (Guiso et al. 2008). In many cases, investment opportunities are analyzed based only on the project information that is available online. Therefore, it is difficult for lenders to ensure the authenticity and integrity of borrowers’ information (Klafft 2008a, b). It is also difficult for them to know whether they are dealing with a legitimate fund seeker. Although lawmakers are developing regulations in many countries, the overall outcome of these efforts is not yet clear.

The P2P literature states that P2P investors can infer the creditworthiness of borrowers by observing the lending decisions taken by other P2P investors (Zhang and Liu 2012). Assuming acquainted investors have an information advantage in a system with posted prices, the decision to invest first could signal the quality of the project to unacquainted investors, who thus develop feelings of trust toward the project. In turn, unacquainted investors trust and expect to benefit from the monitoring capabilities and reciprocal insurance created by acquainted lenders who finance a larger share (Lee and Persson 2016). In addition, more financing from acquainted investors can offer a proxy of social network strength, which can enhance firm performance (Gronum et al. 2012). Borrowers’ reputation is another important factor influencing trust in P2P lending projects. Developing a reputation based on telling the truth and using transparent disclosure can benefit borrowers now and in the future (Michels 2012). Borrowers with a better performance history are more likely to obtain loans and to do so at a lower cost. Thus, lenders use the reputation of borrowers as a signal in their lending decisions. In P2P loans, an effective reputation mechanism can discipline borrowers’ behavior, reducing the probability of default (Ding et al. 2019).

### Social capital

Social capital plays a vital role in mitigating information asymmetries because it can avoid potential inefficiencies triggered by information imbalances, thereby improving market efficiency (Durlauf and Fafchamps 2005; Cassar et al. 2007). According to Putnam (1995, p. 67), social capital relates to “features of social organization such as network, norm, and social trust that facilitate coordination and cooperation for mutual benefit.” Nahapiet and Ghoshal (1998, p. 243) defined social capital as “the sum of the actual and potential resources embedded within, available through, and derived from the network of relationships possessed by an individual or social unit.” Under this definition, social capital has three dimensions: cognitive (shared language), relational (social trust), and structural (the presence or absence of social interaction ties between individuals). In P2P platforms, borrowers and lenders rarely know each other in person, so the third dimension is less applicable than the first two.

The characterization of group-level social capital has major implications for understanding the role of social capital in online P2P lending. The concept of group-level social capital varies depending on the study perspective. From the insider’s perspective, social capital refers to a common asset accessible to all members (Coleman 1990), whereas from the outsider’s perspective, it refers to the process by which individuals within the group use mutual recognition and support to emulate a privileged group where different kinds of capital are included (Bourdieu 1984). Authors have analyzed the factors that influence lending outcomes by studying how the borrower’s group reliability and verifiability can improve (or fail to improve) funding performance. The role of contextual features such as the institutional environment has also been considered. In this sense, the right institutional environment can lead social capital to enhance online P2P lending performance by improving community solidarity (Chen et al. 2016). In addition to improve lending performance (Cassar et al. 2007), social capital has several benefits such as complementing credit information for specific borrowers (Lin et al. 2013), increasing the knowledge-sharing behavior of participants in a virtual context (Chang and Chuang 2011a, 2011b), and facilitating participants’ access to valid information (Birley 1985). Although the benefits seem to be clear, authors have explored both the pro and the con arguments regarding social capital, concluding that both points of views require analysis (Light and Dana 2013). From a conventional point of view, social capital entails trusting reciprocal relationships (Mustafa and Chen 2010). However, too much social capital can lead to protecting mediocrity (Light 2010) and imposing mental conformity to entire groups (Aldrich and Kim 2007), among other detrimental outcomes.

Despite the increasing dependence on group social capital to reduce the uncertainty and risks derived from the fast-paced evolution of online platforms, the P2P lending marketplace is characterized by inaccurate and uncertain information due to anonymity and ubiquity. This social capital may deceive potential lenders and lead them into the wrong lending choices, which can harm their economic performance. Therefore, it is important to find a suitable signaling feature to help prospective lenders. When there is uncertainty surrounding a project, social capital, others’ early contributions, and narratives can help crowdfunders’ make decisions by reducing information asymmetries (Herzenstein et al. 2011; Lin et al. 2013; Moss et al. 2015). These factors represent signals of trustworthiness that trigger herding behaviors (Skirnevskiy et al. 2017; Zhang and Liu 2012).

One important question is how social capital influences the formation of the behavioral biases that affect both individuals’ decisions and P2P lending market performance. These biases include local bias, which represents a deviation from rational benchmarks and occurs when investors’ decisions are biased toward local assets such as local firms or borrowers (Ofir and Wiener 2016; Hirshleifer 2001). This behavior is justified by the idea that greater geographic proximity means a lower risk of default probability (Karlan 2007) due to more active group monitoring (Hung 2006). For external lenders, the support of members of the nearby geographic group is interpreted as a powerful and encouraging signal. Recently, authors have found that local biases are commonly present in the P2P lending market (Jiang et al. 2020). They have tried to determine whether loans attracting local lenders perform better or worse than others. Decisions based on local biases have been found to lead to higher default risk, lower recovery rates, and lower realized return. These findings reflect worse market performance. Based on social capital theories, social capital seems to play an important role in forming local bias because it seeks to facilitate coordination, collaboration, and cooperation, providing mutual confidence among individuals.

#### Social networks

Social capital emerges when individuals are connected to each other. These connections have positive advantages for the individuals and their communities (Portes 1998). Greater complexity and uncertainty in the business environment has led to a focus on social networks (Mohrman et al. 1995). The literature generally focuses on analyzing the optimal network configuration instead of the embedded context of the social network, which significantly affects the role of social capital (Leyden 2003). In an online P2P lending market, there are two main types of social networks: friendship and group networks. There are important differences between the two. Whereas friendship networks mostly represent strongly embedded relationships created outside the objective online economic context (Durlauf and Fafchamps 2005; Lin et al. 2013), group networks are created in online environments with anonymous members between whom there is no interaction. In online P2P lending markets, the main differences relate to the absence or presence of physical connections between individuals and the motivation to create such networks (Putnam 1995). Several authors have studied which type of social network has the best economic performance. Lin et al. (2013) argued that friendship networks can provide better economic performance due to the social stigma cost of default. Other authors have concluded that a lack of connection, recurrent interaction, and closed structures may complicate the development and preservation of social capital by group networks (Nahapiet and Ghoshal 1998; Wasko and Faraj 2005). This process can in turn hinder the achievement of positive lending outcomes. Thus, an effective way to enhance social capital is through the establishment of obligations, norms, and sanctions (Knowles 2006). One problem could be that the constraints that shape human interactions in the P2P lending market (North 1990) are fast evolving but are far from mature (Chaffee and Rapp 2012). Therefore, an emerging question is how best to define an online P2P lending market.

#### Communication

Online marketplaces have allowed P2P lending projects to spread useful signals of value for tangible or intangible contributions through different channels. Communication channels can influence how signals reduce information asymmetries through signal quantity (Schrammel et al. 2009), quality (Brown and Hillegeist 2007), or interpretation (Sunder 2003; Venkat et al. 2004), as well as the scattered experiences and limited attention of receivers (Hong and Stein 2007). Evaluating the suitability of communication channels involves analyzing the way in which each channel operates, which can affect information processing (Wicks 1992) and the credibility of the source. A signal is effective if it is reliable, which often leads to a cost of generating the signal, including delicate aspects such as the “economic cost of dishonesty” (Piñeiro-Chousa, et al. 2016). Furthermore, high levels of credibility can serve as a signal that allows one party to select another one from a long list of signalers (Vismara 2017).

In reference to channels and communication processes in online P2P lending, the key is to provide a mechanism to attenuate information asymmetries around loans offered on the platform. Platform owners must seek to manage the level of information asymmetries in their P2P environments to create more balanced marketplaces and improve the ability of P2P participants to process information about their online transactions (Caldieraro et al. 2018).

### P2P lending risks and returns

The number of transactions made on P2P lending platforms has increased substantially in recent years. The P2P lending industry is a fast-growing financial market. P2P lending platforms such as Lending Club and Prosper have websites that encourage individuals to lend to projects and invite researchers to analyze the transaction process (Bachmann et al. 2011; Klafft 2008a, b; Serrano-Cinca et al. 2015). The first key idea is that two main actors participate in P2P lending transactions: borrowers, who seek money for diverse purposes, and lenders, who lend money to obtain a return (Zhao et al. 2016). Although both participate in the same project, their decision-making perspectives differ greatly (Wu and Hsu 2012). The second key idea relates to the applications (“listings”) submitted by borrowers. Lenders can invest whatever amount they want in these listings, causing two possible outcomes. If the money received by a listing achieves its goal, then it becomes a loan and the funding process is finished (Guo et al. 2016). Conversely, if the money received by a listing does not reach its goal, then the process is also finished, although the intended goal is not achieved. Therefore, lenders have two main tasks: first, to select the loan, and second, to decide on the amount of money to invest.

To help lenders select the best loans, P2P platforms provide rating-based models. These models evaluate the level of risk of loans or the probability of default of borrowers. There are several methods to reduce the risk and probability of default (Bastani et al. 2019). Credit scoring methods are used to rank loans based on their expected probability of default, enabling lenders to minimize investment risks by funding the highest scoring loans (Guo et al. 2016; Malekipirbazari and Aksakalli 2015; Serrano-Cinca et al. 2015). Riskier loans have higher interest rates, so lenders can earn more by funding these loans, as long as borrowers pay. The payment structure (amounts and deadlines) of the loan is of interest for lenders because it makes it easy to measure borrowers’ profitability in P2P lending transactions. Serrano-Cinca and Gutiérrez-Nieto (2016) proposed the internal rate of return as a measure of loan profitability and as an effective interest rate. Therefore, lenders should select loans with the highest internal rate of return. Using deep learning, authors have developed deep, dense convolutional networks (Kim and Cho 2018). Combining hard and soft information, other authors have proposed the examination of descriptive text in loan applications and other borrowers’ historical information through topic modeling in conjunction with a classifier (Jiang et al. 2018). Recently, a five-fold cross-validation method, with six classification performance measurements, was used to discriminate between the best algorithms (logistic regression, artificial neural networks, and linear discriminant analysis) and the worst methods (k-nearest neighbors, classification, and regression tree) for default prediction in P2P social lending (Teply and Polena 2020).

Together with the risk of investment failure, lenders must evaluate loan returns (Serrano-Cinca and Gutiérrez-Nieto 2016). However, score-based models do not allow for the evaluation of risks and returns jointly (Finlay 2008). Faced with the two seemingly conflicting goals of minimizing risks and maximizing expected returns (Deb et al. 2011), lenders do not have to fund an entire loan. Instead, they can participate in different loans. Thus, they face a multi-objective problem, under traditional portfolio optimization theory (Markowitz 1952). Based on portfolio optimization, Malekipirbazari and Aksakalli (2015) showed that accounting for nonlinearity in the learning process improves default prediction. They proposed an instance-based model to predict the return and risk rates of loans in P2P lending. Similarly, Guo et al. (2016) proposed an instance-based model to estimate the risks and returns of loans based on historical data. Cho et al. (2019) used multiple regression analysis to provide an instance-based entropy fuzzy support vector machine model for P2P lending investments. Zhao et al. (2016) were the first to evaluate loans from a multi-objective viewpoint. They reported that lenders seek to meet multiple objectives such as nondefault probability, fully-funded probability, and winning-bidding probability. Two portfolio optimization strategies based on weighted objective optimization and multi-objective optimization were established for selecting lenders’ portfolios. Bastani et al. (2019) proposed a two-stage scoring approach. Loans go from stage one to stage two depending on the probability of default prediction, measured as the internal rate of return through wide and deep learning.

According to Zhang et al. (2020), the effectiveness of the existing credit-score models can be questioned because data complexity can lead to poor classifications. Moreover, these models must be trained and updated online to adapt to scenarios where P2P loan data grow rapidly and change frequently. They advocated new credit-scoring models based on data mining and machine learning (e.g., gradient boosting decision trees or neural networks). These models enable online training and updating and can handle multiple types of features. Babaei and Bamdad (2020) used artificial neural networks and logistic regressions to formulate investment decision making in P2P lending as a multi-objective portfolio. They thus estimated both the probability of default and the return of each loan.

## Method

This section describes the search for scientific publications on P2P lending from 2003 to February 2021. Different bibliometric methods were used.

### Data set

The Web of Science (WoS) database was used to search for publications on P2P lending. In addition to covering other types of publications, the WoS comprises the highest number of papers published in JCR-indexed journals. In comparison with other databases, it provides a large number of high-quality publications with high-quality content (Ball and Tunger 2006; Scaringella and Radziwon 2018). The WoS database was the only source used in this study. This approach is common in bibliometric studies and is advisable so that the data can be handled in a reliable and consistent way. The search engine of the WoS database uses Boolean operators (OR, AND, etc.). The search string used in the WoS Core Collection was TOPIC: (“peer to peer lending”) OR TOPIC: (“crowdlending”) OR TOPIC: (“P2P lending”) OR TOPIC: (“peer to business lending”) OR TOPIC: (“P2B lending”) OR TOPIC: (“business to business lending”) OR TOPIC: (“B2B lending”) OR TOPIC: (“crowd lending”) OR TOPIC: (“crowd-lending”) OR TOPIC: (“peer-to-peer lending”) OR TOPIC: (“peer-to-business lending”) OR TOPIC: (“business-to-business lending”) OR TOPIC: (“peer-to-peer (P2P) lending”).

To obtain accurate results, only documents from the following categories were considered: Economics; Business finance; Business, Computer science information systems; Management; Computer science interdisciplinary applications; Operations research management science; and Social sciences interdisciplinary. To include all published documents on the study topic, no restrictions were established in relation to type of publication, year of publication, or language. As a result, information on 429 studies published from 2003 to February 2021 were downloaded, including the title, keywords, abstract, source, and information regarding the authors and references cited in each publication. A data cleansing process was applied (Zupic and Čater 2015). Microsoft Excel was used for the initial analysis. VOSviewer software was then used to process and analyze the data.

### Sample description

**Fig. 1:**
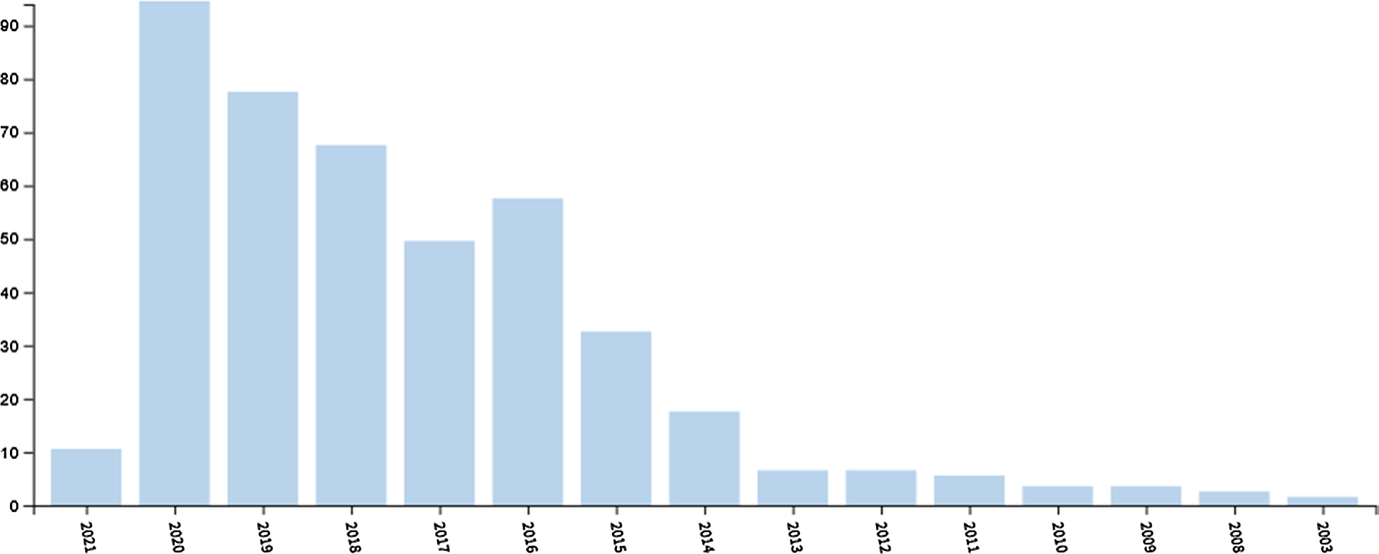
Number of published articles. Source: WoS, retrieved March 5, 2021

Figure 1 shows the number of articles on P2P lending published each year. The study was carried out in February 2021 and the search did not return any papers from this year. The first document dates back to 2003. There was a constant but slow increase in the number of articles published from 2003 to 2013. Since 2014, there has been a substantial increase in publishing activity, especially from 2018 onward. In 2020, more than 90 papers were published on this topic. This result suggests that this research field is current and relevant. The relevance of this field motivated this research.

The categories with the most published papers are Economics, Finance, and Business. The number of papers published in the Management and Computer science categories is also high. This high number of publications reflects the importance of technology in revolutionizing finance, including P2P lending. Although some of the most cited papers in this research field are purely finance-oriented studies, some are related to behavioral finance. This finding reflects the importance of behavioral finance research today (López-Cabarcos et al. 2020) and the massive implications it has in explaining modern forms of financing (Table 1).

**Table 1:** Main WoS categories and most cited papers

WoS Categories	Number of records	% of total
Economics	109	25.41
Business finance	105	24.48
Business	104	24.24
Computer science information systems	93	21.68
Management	90	20.98
Computer science interdisciplinary applications	40	9.32
Operations research management science	39	9.09
Social sciences interdisciplinary	26	6.06
Computer science theory methods	23	5.36
Engineering electrical electronic	23	5.36

Article	Author	Citations
Judging borrowers by the company they Keep: friendship networks and information asymmetry in online peer-to-peer lending	Lin et al. (2013)	333
Rational herding in microloan markets	Zhang and Liu (2012)	264
Trust and credit: the role of appearance in peer-to-peer lending	Duarte et al. (2012)	261
New financial alternatives in seeding entrepreneurship: microfinance, crowdfunding, and peer-to-peer innovations	Bruton et al. (2015)	235
What’s in a picture? Evidence of discrimination from prosper.com	Pope and Sydnor (2011)	218
Evaluating credit risk and loan performance in online peer-to-peer (P2P) lending	Emekter et al. (2015)	155
Strategic herding behavior in peer-to-peer loan auctions	Herzenstein et al. (2011)	146
Home bias in online investments: an empirical study of an online crowdfunding market	Lin and Viswanathan (2016)	139
Herding behavior in online P2P lending: an empirical investigation	Lee and Lee (2012)	134
Tell me a good story and I may lend you money: the role of narratives in peer-to-peer lending decisions	Herzenstein et al. (2011)	125

*Source*: Authors based on WoS data

An item can belong to several categories

### Bibliometric analysis

Bibliometrics refers to the quantitative study of bibliographic resources (Pritchard 1969). Following a systematic literature review (Kraus et al. 2020a, b), science mapping aims to identify the structural and dynamic features of a research field (Noyons et al. 1999) by identifying networks of elements (documents, authors, journals, and keywords) based on their relatedness and classifying them into different clusters (Zupic and Čater 2015). Co-citation analysis (Small 1973), the most frequently applied method, uses the reference set of publications in a database to identify its intellectual structure. Bibliographic coupling (Kessler 1963) is used to study documents that share a common reference. The research frontier of a given field can thus be identified. Keyword co-occurrence (Callon et al. 1983) considers the most frequently used keywords.

This study used these three methods to map the state-of-the-art of P2P lending research. Co-citation analysis of authors and references was used to identify the most relevant authors and studies on P2P lending (Boyack and Klavans 2010). Bibliographic coupling with sources was used to identify the most important journals that publish papers in this research field (Zupic and Čater 2015). Both techniques provided an overview of the past (co-citation) and present (bibliographic coupling) of this research area (Kovács et al. 2015). Finally, keyword co-occurrence was used to determine the core of the P2P lending research field (Su and Lee 2010). Keyword co-occurrence analysis studies the frequencies of specific words that are jointly mentioned (Kraus et al. 2020a, b).

VOSviewer software (van Eck and Waltman 2010) version 1.6.16 (CWTS 2020) was used for the analysis. The three analyses provide networks using maps formed by nodes and links. These nodes and links are grouped into nonoverlapping clusters. Authors, publications, journals, and words are the nodes, and the co-occurrences between them are the links. The size of a node represents the number of connections to other nodes. The closer two nodes are to each other, or the thicker the line that links them, the stronger the connection is between them (Waltman and van Eck 2019). The fractional counting option (Perianes-Rodriguez et al. 2016) was chosen in all analyses performed with VOSviewer software.

With VOSviewer software, it is possible to complement the visual interpretation with tables. These tables can be employed to analyze key metrics for each network, such as density (number of links in relation to the total potential number of links in the network) and degree (average number of links of the nodes in the network). A higher density and a higher degree reflect a more interrelated network (Arho 2019; Vogel and Güttel 2013). This software is useful for this kind of research because it provides a map based on a co-occurrence matrix. This map can be created following a three-step procedure: (1) compute a similarity matrix based on the co-occurrence matrix; (2) build a map by applying the VOSviewer mapping technique to the similarity matrix; and (3) translate, rotate, and reflect the map (Van Eck and Waltman 2010).

## Results

The main results of the analysis are presented in this section. The key articles in this research field were mapped using co-citation analysis, bibliographic coupling, and keyword co-occurrence.

### Co-citation analysis

Table 2 displays the top 10 results for the co-citation analysis of references and authors. The analysis of references reveals the basis of a specific research field. In this case, the analysis shows whether P2P lending studies are purely financial or adopt a behavioral finance perspective. The link strength measures the intensity of the linkages between references or authors. Based on this link strength, the results are consistent with those for the number of citations. The key references are Lin et al. (2013) and Duarte et al. (2012). The most important authors are M. Lin and M. Herzenstein.

**Table 2:** Reference co-citation and author co-citation results

Reference co-citations				Author co-citations		
Title	Author(s)	Citations	Link strength	Author(s)	Citations	Link strength
Judging borrowers by the company they keep: Friendship networks and information asymmetry in online peer-to-peer lending	Lin et al. (2013)	144	136	Lin, M	177	169.2
Trust and credit: The role of appearance in peer-to-peer lending	Duarte et al. (2012)	107	102	Herzenstein, M	165	156.17
What’s in a picture? Evidence of discrimination from prosper.com	Pope and Sydnor (2011)	99	98	Duarte, J	113	112.69
Evaluating credit risk and loan performance in online peer-to-peer (P2P) lending	Emekter et al. (2015)	86	77	Freedman, S	106	98.75
Herding behavior in online P2P lending: An empirical investigation	Lee and Lee (2012)	80	74	Pope, D.G	103	102.6
Rational herding in microloan markets	Zhang and Liu (2012)	63	60	Iyer, R	98	96.21
Tell me a good story and I may lend you money: The role of narratives in peer-to-peer lending decisions	Herzenstein et al. (2011)	62	62	Lee, E	87	85.33
Instance-based credit risk assessment for investment decisions in P2P lending	Guo et al. (2016)	61	47	Emekter, R	86	85.00
Strategic herding behavior in peer-to-peer loan auctions	Herzenstein et al. (2011)	61	58	Serrano-Cinca, C	75	71.04
Emergence of financial intermediaries in electronic markets: The case of online P2P lending	Berger and Gleisner (2009)	52	48	Zhang, J.J	65	64.00

Source: Authors based on VOSviewer results

**Fig. 2:**
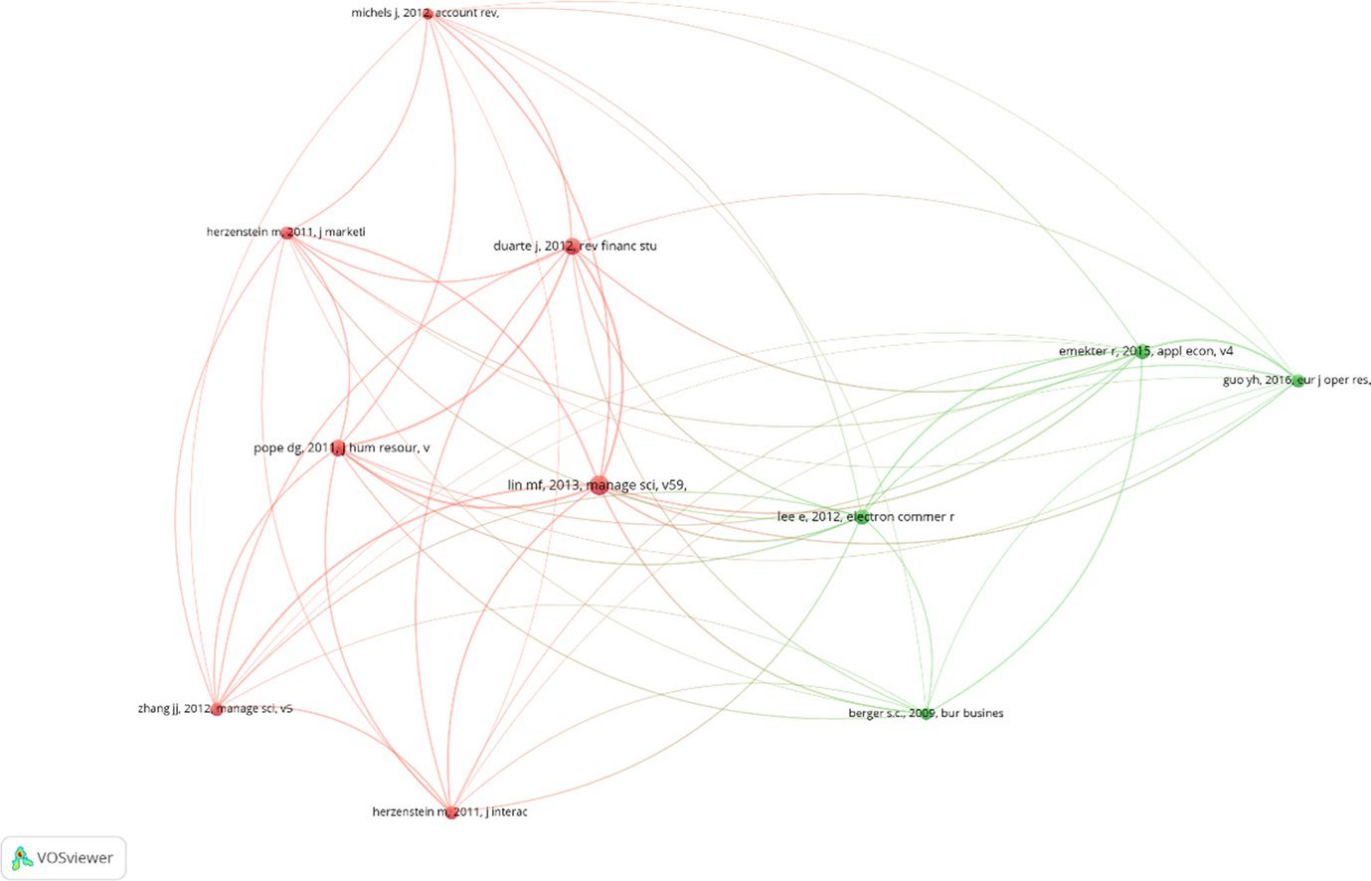
Reference co-citation analysis

Figure 2 presents the top 10 references resulting from the reference co-citation analysis. The minimum number of citations for a reference was set at 50. Of the 10,884 references considered in this study, 11 exceeded this threshold. The number of links was 55 (100% density), the total link strength was 406.5, and the degree was 10. To simplify the analysis of the references network, the references with the highest link strength were selected. A label represents each reference, and the font size denotes the number of times the reference was cited in the database. As mentioned earlier, a larger font size indicates that the reference has been cited more often. The distance between two references represents the probability that these references are cited together. Consequently, shorter distances indicate a higher probability of being cited together. The colors indicate whether there are different clusters of cited references. References in a cluster are more likely to be cited with other references in the same cluster.

The graph contains two clusters. The first (in red) comprises seven papers, primarily oriented to analyzing P2P lending from a behavioral point of view. Specifically, this cluster contains papers on trust or herd behavior (Duarte et al. 2012; Zhang and Liu 2012; Herzenstein et al. 2011). In addition, this cluster contains the most important paper in terms of number of citations and link strength. This paper is located in the center of the network (Lin et al. 2013). The second cluster (in green) comprises four papers that analyze P2P lending from a financial perspective. Lee and Lee (2012) authored the most notable paper in this cluster. Despite forming part of this cluster, it addresses the topic from a financial as well as a behavioral perspective. Moreover, this paper is located very close to the center of the graph. Therefore, it is highly relevant and can act as a nexus between the two clusters. In conclusion, the papers by Lin et al. (2013) and Lee and Lee (2012) are at the core of this field and provide a basis for research in this area.

**Fig. 3:**
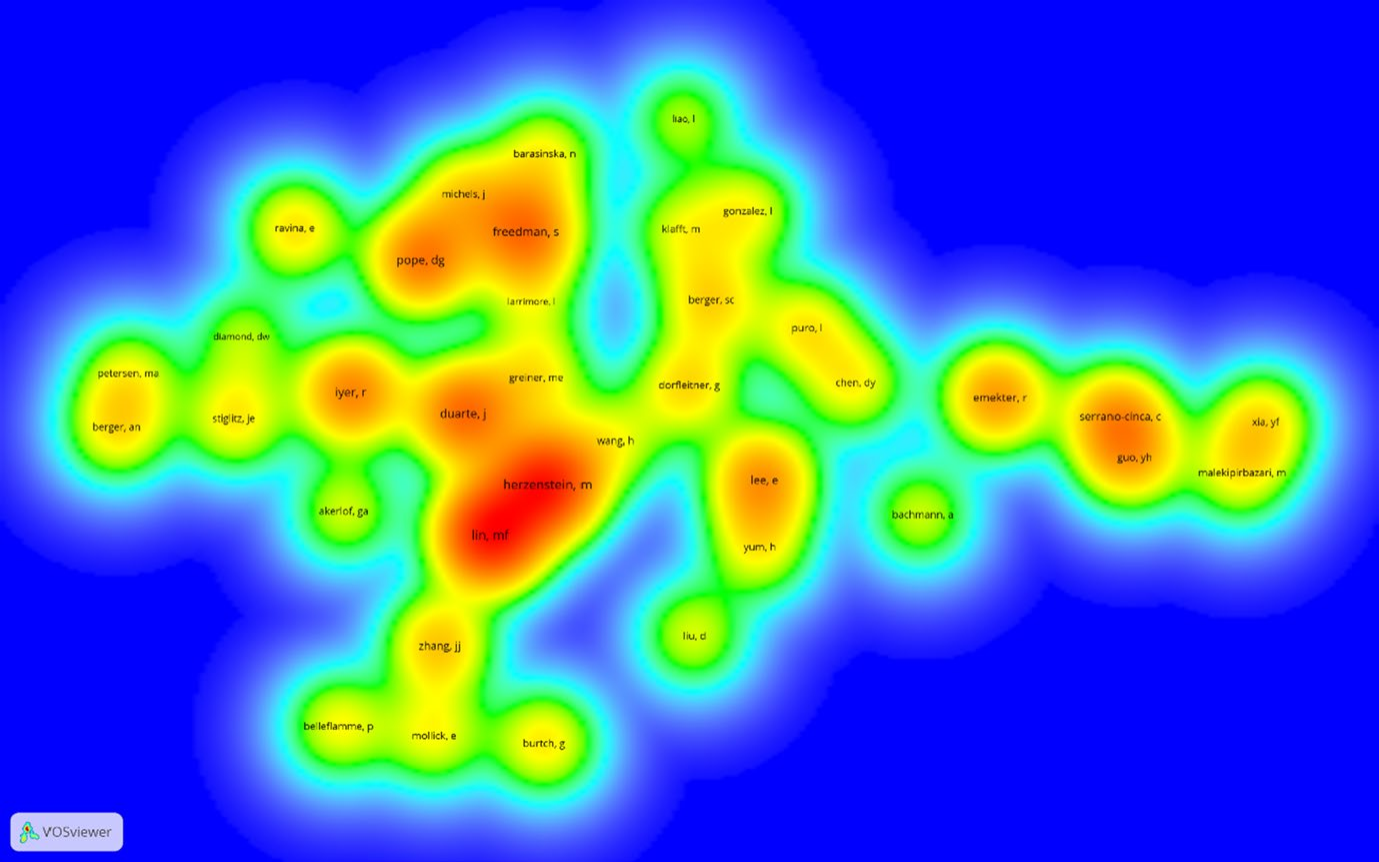
Density map of author co-citation analysis

The author co-citation analysis reveals the most cited authors. These papers are generally among the top 10 most cited references. When considering the link strength of author co-citations, there are some slight differences with respect to the reference co-citation analysis. In this case, although the difference is very small, the author D. G. Pope is slightly more important than S. Freedman. There seems to be no divergence between the results of the author co-citation analysis and the reference co-citation analysis. These results indicate that M. Lin is the most cited author and that a paper by the same author has received the most citations. The other most cited authors are M. Herzenstein and J. Duarte. Figure 3 displays the density map of the co-citation analysis of authors. The minimum number of citations of an author was set at 30, with 39 (out of the 7,773) authors exceeding this threshold. There were 729 links (98% density), a total link strength of 1,124.04, and a degree of 37.4. The possibility that there were different authors with identical names or the same author with different names was checked. Such duplicate entries can greatly affect the results. No discrepancies were found. The most cited authors appear in red. In contrast, authors in green have the lowest number of citations. The position on the map denotes the proximity between authors. The closer they are, the greater the chance is that they are cited together. The map shows three groups of authors. The central group includes the most relevant authors (M. Lin, M. Herzenstein, and J. Duarte). The group located at the top of the map but very near the center is led by S. Freedman and D. G. Pope. These two groups include authors that study P2P lending from a behavioral as opposed to a financial point of view. This finding is consistent with those for Cluster 1 of the co-citation analysis of references. The group on the right includes Y. H. Guo, who analyzes P2P lending from a financial point of view. This finding is consistent with those regarding Cluster 2 of the co-citation analysis of references.

### Bibliographic coupling of sources

To map the journals that publish research on P2P lending, the minimum number of documents of a source was set at five. Of the 283 sources, 11 exceeded this threshold. There were 55 links (100% density), a total link strength of 503.82, and a degree of 10. The minimum number of citations was also set to zero so as not to penalize more recent publications.

Table 3 shows the ranking of journals with the most published papers on P2P lending. The journal with the most publications (15) is *Electronic Commerce Research and Application*. It has the highest link strength (242.19), although it does not have the highest number of citations (390). The journal with the highest number of citations (785) is *Management Science*. It has a moderate link strength (75.78), implying that this journal includes highly cited publications in this field but that the publications are not closely related to the publications in other journals. Different results were observed for *Electronic Commerce Research* and *Journal of Management Information Systems*. They have published few papers on this topic (six and five, respectively, with 42 and 153 citations, respectively). However, their link strengths are quite high (115.23 and 110.15, respectively). Thus, their publications, despite being scarce, are relevant in this field.

**Table 3:** Bibliographic coupling of sources

Source	Documents	Citations	Link strength
Electronic commerce research and applications	15	390	242.19
IEEE access	10	42	97.73
Finance research letters	8	31	71.15
Emerging markets finance and trade	7	14	91.57
Financial innovation	7	44	64.97
Electronic commerce research	6	42	115.23
European journal of operational research	6	275	92.74
Management science	6	785	75.78
European journal of finance	5	3	52.27
Journal of management information systems	5	153	110.15

Source: Authors based on VOSviewer results

**Fig. 4:**
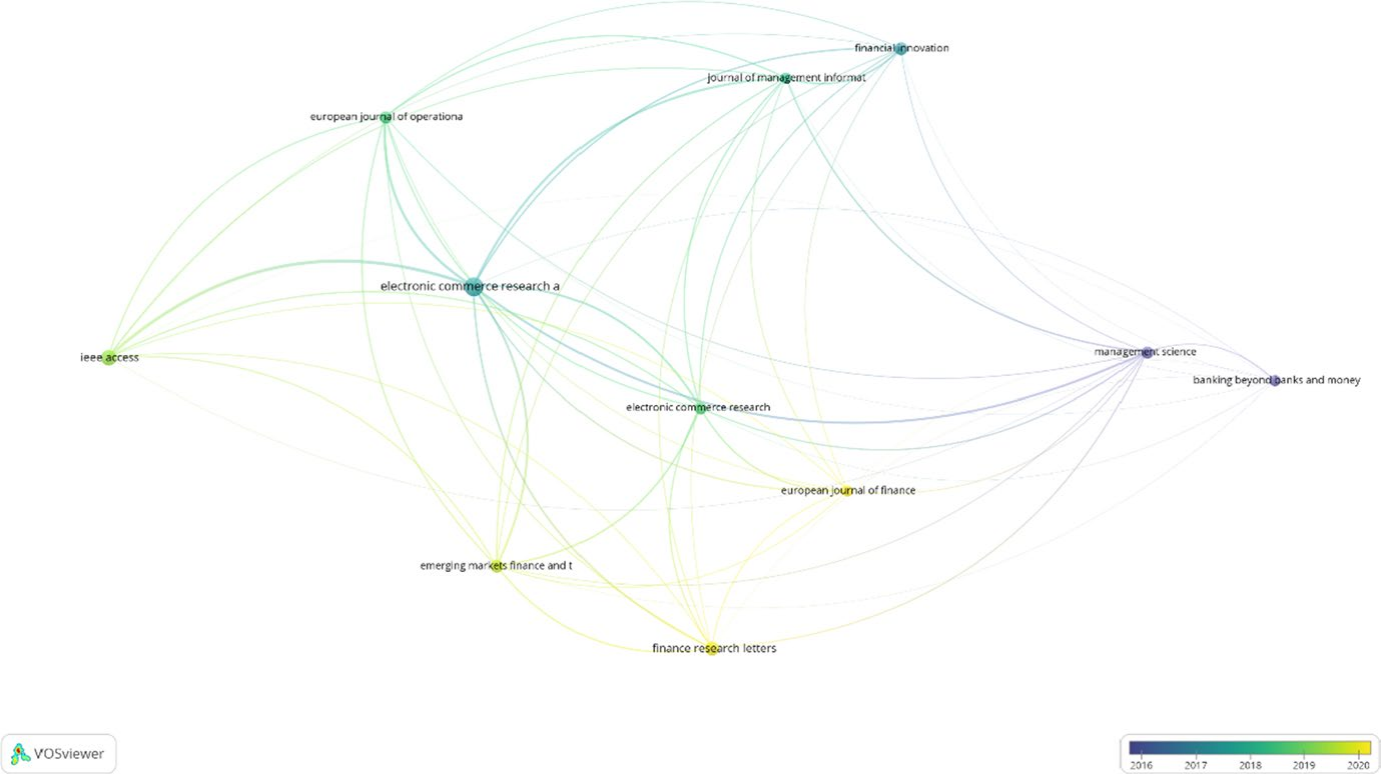
Bibliographic coupling of sources by average year of publication

Figure 4 shows that the journals that published the most papers on P2P lending during the second half of 2019 and 2020 were *Finance Research Letters*, *Emerging Markets Finance and Trade*, *European Journal of Finance*, and *IEEE Access*. *Finance Research Letters* has published the most papers (8) and has the most citations (31). *Emerging Markets Finance and Trade* has the highest link strength (91.57).

### Author keyword co-occurrence

**Fig. 5:**
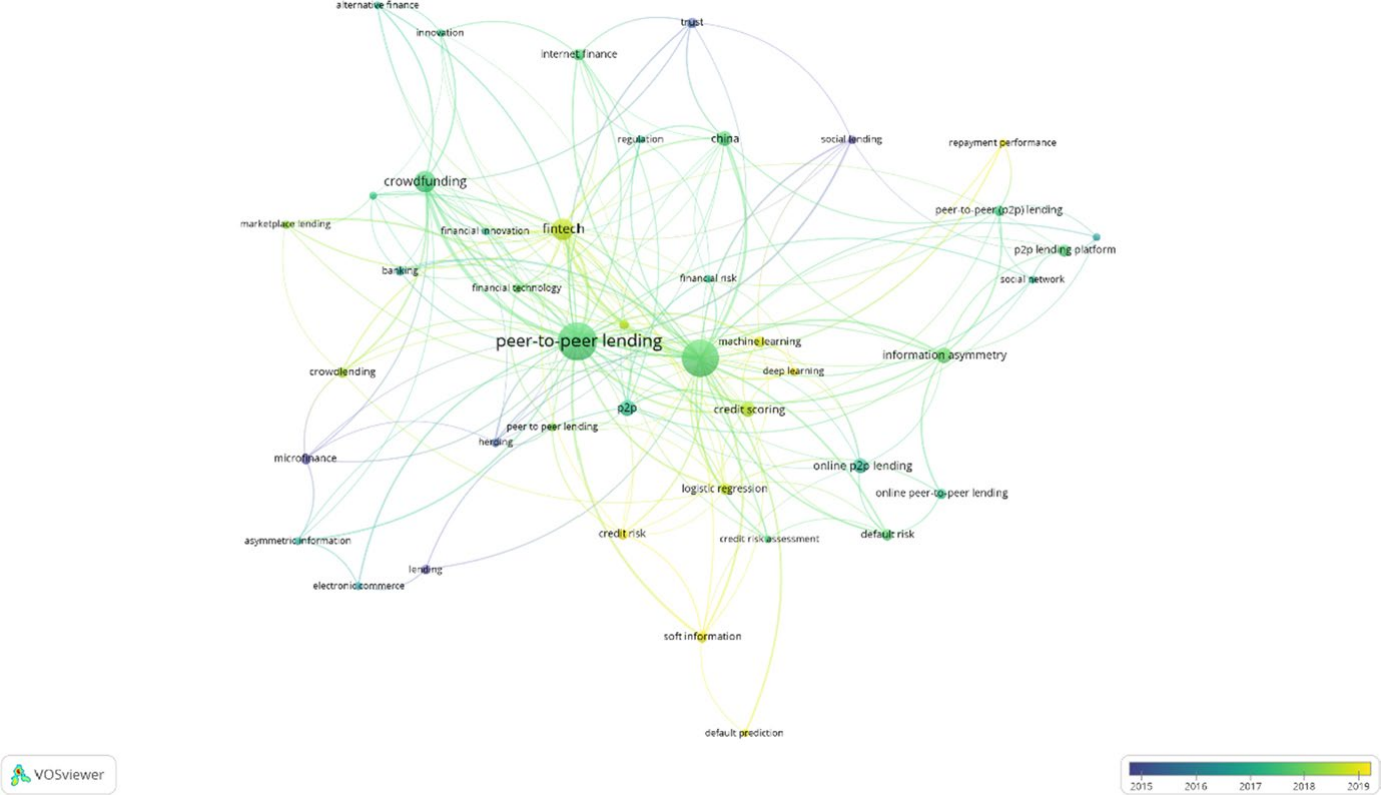
Author keyword co-occurrence by average year of publication

Figure 5 shows the average year of publication of the documents with an author keyword. A threshold of five occurrences was used. Of the 1,131 considered, 43 keywords exceeded this threshold. The network is quite scattered, with 199 links (density of 22%) and an average number of links per keyword (degree) of 9.3. The results indicate that the most common keyword (107 occurrences) is “peer-to-peer lending”. This keyword has the largest circle in the graph (link strength 60.00). This keyword appears in green at the center of the graph, which means that the documents containing this keyword were mostly published between 2017 and 2018. The next most important keyword is “P2P lending” (with 103 occurrences and a link strength of 59), followed by “crowdfunding” (with 36 occurrences and a link strength of 30), “fintech” (with 35 occurrences and a link strength of 30), “information asymmetry” (with 18 occurrences and a link strength of 15), “P2P” (with 18 occurrences and a link strength of 10), and “online P2P lending” (with 17 occurrences and a link strength of 4). These results appear in Table 4. This analysis reveals the recent appearance of the keywords “fintech”, “machine learning”, “deep learning”, and “soft information. These keywords reveal the trend toward innovative finance based on new technologies such as blockchain, big data, and artificial intelligence. This conclusion is consistent with the results of the bibliographic coupling analysis with sources, which include technology-oriented journals such as *Electronic Commerce Research and Applications*, *IEEE Access*, *Financial Innovation*, and *Journal of Management Information Systems*. This result is also consistent with the WoS categories considered in the analysis, where the categories Computer science information systems, Computer science interdisciplinary applications, and Computer science theory methods are found to be relevant in this area.

**Table 4:** Author keyword co-occurrence

Keyword	Occurrences	Link strength
Peer-to-peer lending	107	60.00
P2P lending	103	59.00
Crowdfunding	36	30.00
Fintech	35	30.00
Information asymmetry	18	15.00
P2P	18	10.00
Online P2P lending	17	4.00
China	16	14.00
Credit scoring	16	14.00
P2P lending platform	11	4.00

*Source*: Authors based on VOSviewer results

## Limitations and practical implications

Bibliometric analyses are subject to several limitations. Some limitations relate to the own application of the bibliometric techniques. For example, not always exists conceptual or methodological ‘proximity’ between publications that are jointly cited, as co-citation analysis proposes; or not always the number of citations means importance or relevance of a work, since citations can be the result of many factors influencing researchers when writing their papers. A deep review of the publications considered in the study in the first case, and the use of citations patterns considering both total citations and citations excluding authors’s self-citations in the second case, can help solve these limitations. Other common limitations are related to the method and choice of data. For example, although the search terms in this study were carefully selected, a broader or more restricted set of publications would have been obtained if other search terms had been considered. These differences would have influenced the study results. Moreover, some documents may have been overlooked because a single citation database was used. For example, the most up-to-date references that have not yet been assigned to a specific topic are not included in the WoS. In future studies, other databases (e.g., Scopus and Google Scholar) could be used to compare the findings with those from this study or simply to include a greater number of publications. All the analyses were performed using the most objective criteria possible. However, a certain degree of subjectivity is inevitable. For example, decisions must be taken when deciding on the number of authors or references to include in the analyses. Moreover, the author co-citation analysis only considered the first author of each document. Therefore, information on collaborating researchers was lost (Córdoba-Cely et al. 2012). However, this procedure is common in bibliometric studies. Finally, the analysis of authors considers their affiliations at the time of publication, which can lead to discrepancies. So, to avoid discrepancies and ensure exact correspondence between the authors’ names in the database and those in the publications, a thorough search and correction process was performed. Despite these limitations, this paper still offers valid analysis of the most relevant research on P2P lending over the study period. This analysis in turn provides a structural and dynamic overview of the research field.

A comprehensive set of the most relevant publications on P2P lending from 2003 to February 2021 was analyzed. By examining the research frontiers in the field of P2P lending and assessing the extent to which this field can form a new and independent area of research, this study has important implications for researchers, firms, and policymakers. Researchers can build a picture of this novel research field. This picture can help them identify its core theoretical framework as well as the key topics that show the directions that research should take in the future. For example, innovative finance based on new technologies such as blockchain, big data, machine learning, and artificial intelligence is the most novel trend in this field. Moreover, managers, entrepreneurs, and all kinds of companies, especially those involved in P2P lending projects, can use this research to find evidence of the factors, advantages, weaknesses, and challenges affecting them. Finally, finding and applying theoretical and practical knowledge of P2P lending transactions can help policymakers target their efforts and design policies and programs that contribute to the effective and ethical functioning of the P2P lending market.

## Conclusions and future research

Three bibliometric techniques were applied to WoS-indexed publications from 2003 to February 2021. The aim was to describe the knowledge frontier in P2P lending research. The analyses reveal the following: (1) the main P2P lending research topics, (2) the consolidation of P2P lending as an independent research field, (3) the strong interconnectedness between the most common P2P lending research topics, and (4) the early development of the P2P lending research field.

An even greater technological revolution will surely take place in the financial sector. Therefore, alternative financial markets and new ways of understanding finance will continue to appear and to expand in the near future. In this revolution, the role of each agent must be clearly established. The changes that take place will reshape and influence the investment philosophy, behavior, and decision-making processes of investors and the expectations and motivations of borrowers. In the P2P lending market, decisions must be made considering information related to market trading volume, posted interest rates, monetary returns or default probability, and individual behavior associated with investor decisions. The motivations, expectations, and specific circumstances surrounding people are vital to determine the outcome of P2P lending transactions.

The study results suggest that future research on P2P lending should focus on the behavioral component of these transactions. For example, research should answer the question of how individual-related and context-related issues affect P2P lending transactions, considering the perspectives of both borrowers and lenders. Future research should also examine how to reduce information imbalances by avoiding the noise it generates, which can prevent these transactions from functioning correctly. It would also be valuable to analyze the moderating role of some variables (innovation orientation, entrepreneurial orientation of borrowers and lenders, and cultural values) in this causal relationship.

Researchers together with public institutions and organization should analyze the way to develop a framework for the risk management and profitability in the P2P lending market with the aim of generating a greater use and confidence around P2P financial products. Undoubtedly, this synergy could enhance the sustainability and development of the P2P lending market. Furthermore, most of the P2P lending studies are based on qualitative research that seeks to identify the shortcomings and opportunities of this alternative investment and financing formula, so there is a great potential for new quantitative research.

High-tech and decentralized financial environments are destined to prevail over more traditional areas. This scenario requires analysis of the variables that can affect these new financial contexts and decrease risks and the probability of failure. Aspects such as loan maturity, interest rates, platform size, participants’ geographic location, and guarantees to reduce the likelihood of default must be analyzed in depth. One aspect that deserves special attention is the situation of information asymmetry that arises in P2P lending transactions. Information imbalances can greatly influence P2P lending outcomes, so mechanisms should be used to avoid or at least minimize such imbalances. Aspects derived from the concept of social capital or the disclosure of accurate information to the P2P lending market are two effective mechanisms to avoid both information asymmetries and the probability of default.

As the fourth industrial revolution takes hold, some questions must be answered. One key question is how to benefit from this revolution in new financial environments. Many countries and firms around the world are designing policies and making strategic decisions to promote technology-based financial environments, which seem to be an essential part of the current and future economic context. The regulations established by governments and other authorities must keep up with the appearance of new financial products, services, and markets. This need to keep pace does not mean that regulations should hinder technological advances or innovation. However, authorities must use these regulations effectively to develop reasonable rules to guarantee a healthy future of the financial industry and protect the rights of all participants. Thus, there is an urgent need to educate individual investors (considering variables such as gender, age, and previous education) and design a valuable financial ethos capable of promoting a values-based foundation that benefits all areas of society.
